# Added value of cardiac deformation imaging in differential diagnosis of left ventricular hypertrophy

**DOI:** 10.21542/gcsp.2018.21

**Published:** 2018-08-12

**Authors:** Filip Loncaric, Bart Bijnens, Marta Sitges

**Affiliations:** 1 Cardiovascular Institute, Hospital Clínic, University of Barcelona & IDIBAPS (Institut d’Investigacions Biomèdiques August Pi i Sunyer); 2 Universitat Pompeu Fabra, Barcelona, Spain; 3 ICREA, Barcelona, Spain

## Hypertrophic cardiomyopathies: A heterogeneous group of cardiac involvement

Hypertrophic cardiomyopathy is clinically defined by the presence of increased left ventricular (LV) wall thickness that is not solely explained by abnormal loading conditions.^1^ The aetiology is diverse, but in 60% of patients the disease is an autosomal dominant trait caused by cardiac sarcomere protein gene mutation (HCM). In 10% of cases, the cause is inherited metabolic and neuromuscular disease, chromosomic abnormalities and genetic syndromes, whereas, in the remaining 30% of patients the aetiology remains unknown.^1^

## Challenges of imaging in LV hypertrophy

In everyday clinical practice, contractility and pump function are estimated using indirect measurements with 2D echocardiography that often rely on geometric assumptions where ventricular shape, wall thickness and cavity size directly influence the validity of the results.^6^ The fact that LV hypertrophy is commonly heterogeneous in its distribution in HCM, challenges the use of standard methods for assessing contractile function.

Although LV ejection fraction (EF) and outcome are highly associated in other clinical settings with moderately and severely impaired ventricular function, EF is not such a good predictor of outcome in LV hypertrophy.^7^ Even though systolic function in HCM is often locally impaired, patients often have a preserved or even supernormal EF^6^ as hypertrophic ventricles thicken more in absolute terms, resulting in a greater LV cavity reduction and a high values for EF.^6^ Furthermore, 2D echocardiography alone cannot definitely distinguish myocardial hypertrophy from interstitial infiltration and intracellular accumulation of metabolic substrates.^8^

Other common diagnostic challenges include co-existent pathologies like hypertension and HCM, isolated basal septal hypertrophy in the elderly, physiological hypertrophy caused by intensive training, and presentation in the late phase of the disease with LV wall thinning.^1^ To meet these challenges other diagnostic tools must be utilized, including genetic testing, cardiac MRI, CT and deformation imaging with assessment of strain and strain rate.

### Cardiac deformation imaging

**Figure 1.**
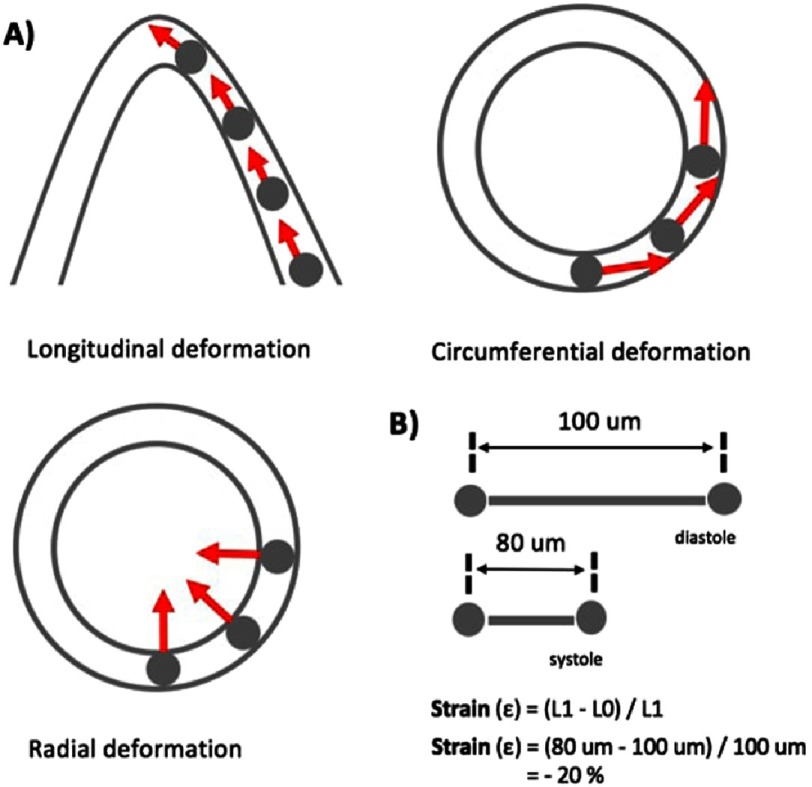
(A) Three main components of cardiac deformation (B) Strain quantifies the myocardial fibre deformation during the cardiac cycle. *Example:* If the initial length of the fibre is 100 µm in diastole and 80 µm after maximal contraction in systole, then the strain value of cardiac deformation (ε) is equal to −20%.

Strain is a percentage change in the length of a myocardial segment that describes regional LV function.^6^ Normal cardiac deformation consists of longitudinal lengthening and shortening, circumferential lengthening and shortening and consequently, radial thinning and thickening (Figure 1A).^9^

**Figure 2.**
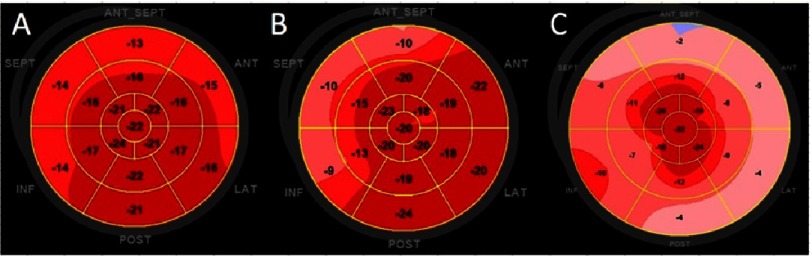
Representative polar maps of peak systolic longitudinal strain (bull’s eye plots). (A) Healthy patient with a relatively homogenous distribution of strain values. (B) Hypertensive patient with septal longitudinal strain impairment. (C) Patient with cardiac amyloidosis and an apical sparing pattern with reduced deformation of the basal and mid ventricular segments.

Peak systolic strain describes the relative length change of the LV myocardium between end-diastole and end-systole. Strain rate is the spatial derivative of local myocardial velocities (strain is the temporal derivative of strain rate)^10^. Strain rate has a strong relationship to contractility but is limited by signal to noise ratio and low frame rate.^6^ In addition to strain and strain rate curves, a colour-coded ‘Bull’s eye view’ of the LV is often used to visualize regional strain values (Figure 2).

Deformation can be quantified with velocity based tissue Doppler imaging (TDI) or grey scale imaging techniques like speckle-tracking echocardiography (STE). STE quantifies the deformation of the myocardium by identifying interference patterns (speckles) generated by acoustic markers in the myocardium in interaction with ultrasound waves. Displacement of these speckles can be tracked frame-to-frame during the cardiac cycle giving information about the deformation of a point in a myocardial region of interest. In each point, strains are calculated in circumferential, longitudinal, and radial directions.^11^

Compared to TDI, STE is more reproducible, less dependent on user expertise and has been validated in comparison with tagged magnetic resonance imaging^10, 12^. Limitations of STE include lack of inter-vendor standardization^13^, dependence on optimal frame rates (50–90 fps) to precisely characterize regional myocardial motion^14^, load-dependency (preload and afterload influence the measurements^15^), and finally, the fact that age and gender represent a source of variation in measurements^16^.

### Deformation imaging in a healthy heart

Longitudinal deformation can be measured and averaged from the three apical views, giving the value of global longitudinal strain (GLS), while the radial and circumferential deformation are measured from the parasternal short axis views giving global radial and circumferential strain. Deformational change is marked as a negative value if the tracked region shortens, meaning that in long-axis views regions with normal longitudinal contraction have a negative strain value (Figure 1B). In short-axis, the LV circumference shortens during systole, whereas the radial thickness increases. Hence the normal circumferential strain is negative, and the radial strain positive. In a meta-analysis including 2,597 healthy volunteers, normal GLS value was set between −15.9% and −22.1%, normal global circumferential strain between −20.9% and −27.8%, and normal global radial strain between 35.1% to 59.0%.^17^

**Figure 3.**
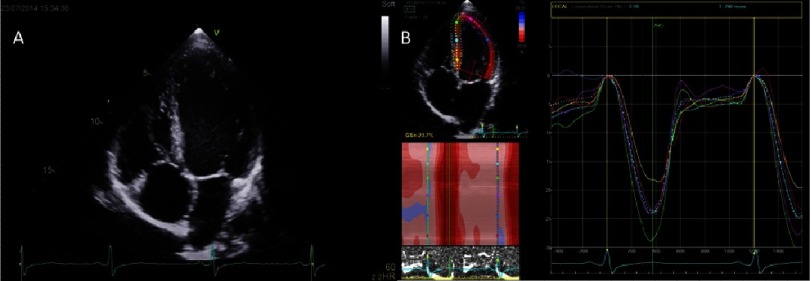
A healthy 21-year-old male. (A) There is no visible thickening of the septum or the free wall in the four-chamber view. Left ventricular cavity and the atria are not dilated. (B) Peak systolic strain curves show all visible segments have normal values and timings of peak-systolic longitudinal strain. Global longitudinal strain is also preserved.

The apical views capture a greater amount of tissue over the length of the entire myocardial wall and the images obtained are of superior resolution - making GLS the most commonly used and robust parameter to quantify cardiac deformation (Figure 3).^12^ If regional tracking is suboptimal in more than two myocardial segments, the calculation of GLS is not recommended.^18^ To reduce variability in measurement special care must be given to obtain good image quality, optimal frame rate, minimize long-axis foreshortening in apical views and ensure perpendicular imaging in short-axis views.

In contrast to LVEF, STE assessment of longitudinal systolic deformation is a more sensitive tool in detecting early systolic dysfunction.^6, 19^ This is partly because of the multi-layered fibre orientation in the myocardium. Subendocardial fibres predominantly determine longitudinal strain, while the midmyocardial and sub-epicardial fibres mainly determine circumferential and radial strain. In numerous cardiac diseases, the sub-endocardium is the first to compromise; therefore, a decrease in longitudinal strain is the earliest indicator of pathology, often with a compensatory increase in circumferential and radial function.^10^ With deformation imaging, it is possible to discriminate between active myocardial segmental deformation and passive displacement of a dysfunctional myocardial segment due to adjacent segment tethering and global cardiac motion.^12^ Most importantly, peak systolic strain is relatively homogeneous in a healthy heart, whereas typical regional strain patterns occur in different cardiac pathologies giving room to added value in diagnostic insight into the underlying disease.

### Hypertrophy as a result of sarcomere protein gene mutations

Genetic mutations of the sarcomere protein genes are inherited by an autosomal dominant pattern, with age-related penetrance and variable expressivity.^20^ The incidence of HCM is presumed to be around 1 in 500 people –making it one of the most common genetic heart diseases. Due to recent advances in genetic population studies, recognition of genotype-positive-phenotype-negative patients, clinical screening of HCM families and enhanced detection of HCM phenotype by advanced imaging, it has been suggested that the incidence may be up to 2.5-fold more common than currently presumed.^2^

The onset of LV hypertrophy varies through life, most often occurring in adolescence, rarely in infants, young children or adults. Hence, in affected families, screening is usually initiated after 10 years of age. Age-related penetrance means that first-degree relatives should be offered follow-up assessment even when there are no definite disease-causing genetic mutations in the proband.^21^

Patients have a variable clinical presentation and natural course. Outcomes include thromboembolism^3^, heart failure^4^, and sudden cardiac death (annual rates around 1%) - yet many patients achieve normal life expectancy. Sudden cardiac death (SCD) is a rare but calamitous outcome, more common in the <30-year-old patient subgroup. With regards to treatment interventions (drug therapy, ICD implantation, heart transplantation), the expected annual mortality rates in adult HCM patients have reached a low 0.5%, as compared to previously expected rates of about 1.5%/year.^5^

HCM caused by sarcomere protein mutations is characterized by disruption of myocardial architecture, cardiomyocyte hypertrophy, fibre disarray and patchy interstitial fibrosis^22^ resulting in increased regional wall thickness, increased LV mass, and impaired cardiac mechanics in hypertrophic segments^23^. As previously mentioned, until the end-stages of the disease, LVEF is mostly preserved or even supernormal in patients with HCM.

**Figure 4.**
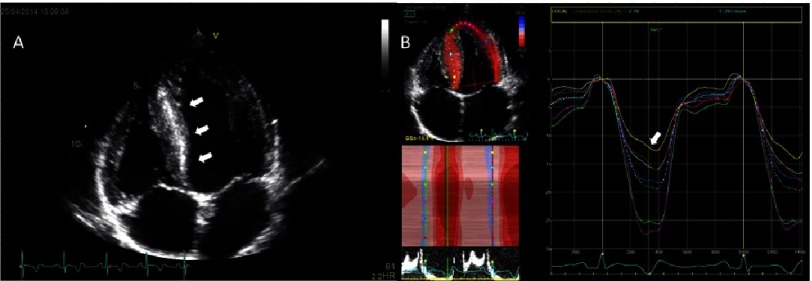
A 20-year-old male football player with sarcomere mutation positive HCM. (A) There is visible wall thickening in the basal, mid and apical inferoseptum (*arrows*) and throughout the anterolateral free wall. There is bi-atrial enlargement, but no enlargement of the LV cavity. (B) There is noticeable reduction of peak-systolic longitudinal strain in the basal inferoseptum (*arrow)*. Global longitudinal strain is preserved.

The greatest reduction in longitudinal strain is seen at the site of hypertrophy, most often in the interventricular septum (Figure 4), while radial and circumferential strain can be preserved.^6^ Strains can be abnormal in mutation-positive patients even before any thickening of the myocardium is seen with conventional echocardiography.^23^ In HCM, the reduction in GLS proved to be an independent factor associated poor cardiovascular outcomes - most strongly with heart failure.^24, 25^

**Figure 5.**
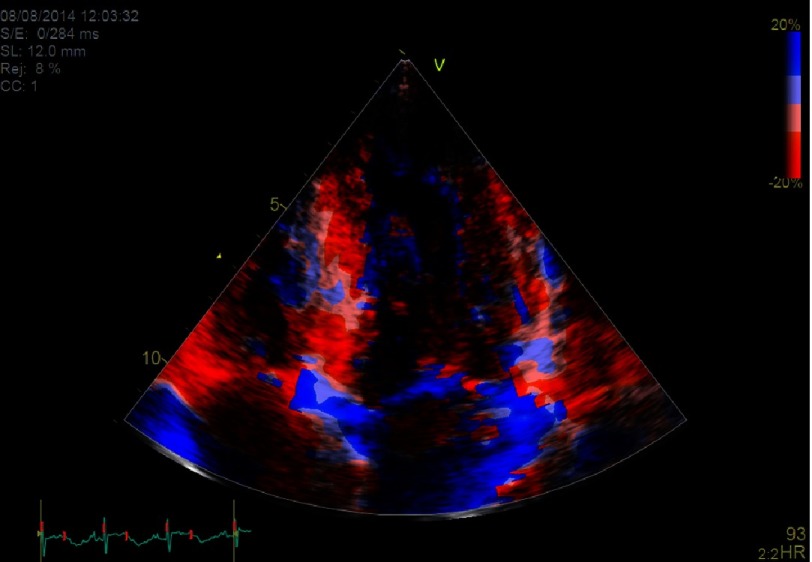
A patient with a sarcomere mutation positive HCM evaluated with color-coded Doppler Tissue Imaging. Different color-codes correspond to different deformation within the hypertrophied inferoseptum. Heterogeneity can be noted throughout the septum, with no deformation in parts of the mid-septum (*light red and blue color*).

Typically, in HCM patients there is a significant heterogeneity in regional myocardial deformation and wall thickness, with areas of normal deformation within areas of abnormal deformation in the same ventricular region, a finding possibly related to the heterogeneous distribution of myocardial disarray in HCM. To evaluate this, the colour-coded Doppler Tissue Imaging is particularly useful as it can readily depict the deformation of myocardial areas within adjacent ventricular segments (Figure 5). Impairment in GLS is also seen in regions with myocardial fibrosis as assessed by CMR late gadolinium enhancement (LGE).

### Hypertrophy as a result of increased afterload: The hypertensive heart

Hypertrophy of the LV in the setting of increased afterload is the response of the myocardium to a continuously increased ventricular wall stress. The pressure inside the ventricle is transferred to the myocardial wall dependent on the local geometry. Because of the variable local radius of curvature of the myocardial walls, the wall stress is heterogeneously distributed on different LV segments. Wall stress triggers the activation of a compensatory mechanism of myocardial remodelling resulting in gradual wall thickening over time.

Arterial hypertension is an example of increased afterload, over time resulting in concentric hypertrophy of the LV coupled with diastolic dysfunction and increased LV filling pressures. The wall thickness in hypertensive hypertrophy is influenced by ethnicity, neurohumoral factors and genetic variants, but in the majority of patients maximal interventricular septal thickness is less than 15 mm.^1^ Unlike HCM, there is no myofibril alignment disarray in regions with hypertrophy. With regulation of blood pressure in hypertensive patients, a regression of LV hypertrophy can be seen, an effect not seen in HCM.

**Figure 6.**
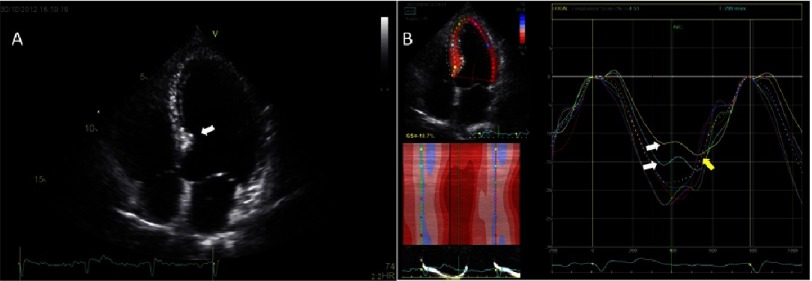
A 44-year-old male patient with arterial hypertension. (A) There is pronounced localized hypertrophy in the basal inferoseptum - septal bulging (*arrow).* The left ventricle and atrium are not dilated. (B) Strain curves show reduced peak-systolic longitudinal strain in the basal and mid-basal inferoseptum (*white arrows)*. Global longitudinal strain is preserved. There is visible post-systolic shortening localized in the basal septum - a typical feature of increased afterload or of ischemic myocardium (*yellow arrow)*.

In cases of high blood pressure, due to the increased local radius of curvature, the basal septal segment is the first to show subclinical systolic dysfunction, quantifiable by changes in deformation - decrease in longitudinal strain (Figure 2B) and typically a post-systolic thickening (Figure 6B).

Post-systolic thickening results from the localized increased wall stress in the basal septum that typically has a reduced deformation and increases deformation when load decreases, after the closure of the aortic valve. These findings can precede the increase in regional wall thickness - also known as ‘septal bulging’ (Figure 6A) – and the impairment of LV EF assessed with 2D echocardiography.^26^ With persistent systemic hypertension or overload, gradually all other segments will develop hypertrophy and will show a decrease in LS.

Hypertensive hearts might ultimately present with a reduced GLS in advanced stages of the disease with persisting poor-controlled hypertension, the magnitude of which is associated with diastolic dysfunction, independent on afterload changes and degree of LVH.^27^ However, in a study comparing patients with hypertensive hypertrophy to HCM patients, a significantly higher GLS was found in hypertensive patients (−17.8 ± 3.1% vs −11.2 ± 4.2%).^28^

### Physiological hypertrophy: Athlete’s heart

The term ‘athlete’s heart’ is often used to describe the adaptation of the heart to long-term physical activity and related haemodynamic overload. In athletes predominantly participating in isotonic or endurance training, a combination of decreased peripheral resistance and increased venous return results in increased preload and chronic volume overload. Over time, these changes lead to eccentric LV hypertrophy with increased wall thickness and LV mass, coupled with proportionally increased LV end-diastolic volumes.^29^ This is in contrast to HCM, where hypertrophy occurs at the expense of cavity size leading to a small LV end-diastolic volumes^6, 29^.

**Figure 7.**
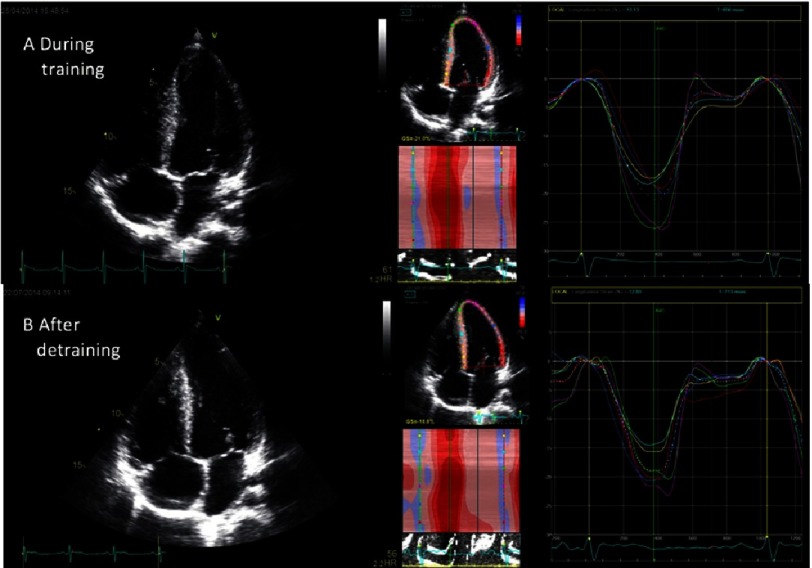
A 16-year-old male athlete with physiological hypertrophy. Due to clinical suspicion of HCM the patient underwent a 3-month detraining period. (A) At admission, the patient had intermediate wall thickening in the inferoseptum and an enlarged left ventricle. All myocardial segments had normal peak-systolic longitudinal strain values and global longitudinal strain was preserved. (B) After the 3-month detraining period, there was a reduction of LV end-diastolic cavity size and a reduction in the dimensions of the inferoseptum. There was no relevant change in segmental peak-systolic longitudinal strain values or the global longitudinal strain.

Current consensus defines the upper limit of physiological adaptation in endurance training to a LV diameter of less than 65 mm and LV wall thickness of less than 14 mm^30^, although 2% of highly trained athletes develop wall thickness between 13 and 15 mm^31^. In some athletes with dimensions above these upper limits there is a phenotypical overlap with lower levels of disease penetrance in HCM, thus defining a grey zone between physiology and pathology. In these cases, a pragmatic method of resolving clinical dilemmas can be applied including a prolonged period of detraining, where reduction of wall thickness may be a sign of physiological hypertrophy (Figure 7). Pellicia et al.^32^ demonstrated that long-term deconditioning in athletes with initial signs of hypertrophy resulted in a 7% reduction in end-diastolic cavity size, though absolute cavity size remained enlarged in 85% of athletes. Wall thickness, initially averaged at 12 mm, showed a 15% decrease, though in 38% there was little or no change. Similar changes in deconditioning are not expected in HCM.^31^

Besides the myocardial morphological adaptations, with the help of deformation imaging, it is possible to identify functional adaptions to intensive exercise and potentially differentiate it from HCM. The exclusion of pathology may be suggested by a finding of normal strain values in the hypertrophied regions - a finding not seen in HCM caused by sarcomere protein mutation.^28^

In a study comparing patients with HCM to professional athletes^28^, patients with HCM had a lower median GLS compared to athletes (−11.2 ± 4.2 vs. −17.8 ± 2.2%). Caselli et al.^33^ evaluated 200 Olympians demonstrating normal GLS and strain rate in highly trained athletes. Even in 5 athletes demonstrating wall thickness of more than 13 mm, there was no difference in GLS or strain rate as compared to the rest of the cohort. Therefore, reduction in GLS is not considered a part of physiological adaption to training.

However, more than the absolute value of GLS, it is important to analyse the distribution and the pattern of myocardial deformation to differentiate true from hypertrophic adaptation to training. Typically, in HCM patients there are areas of normal deformation within areas of abnormal deformation, potentially related to myocardial disarray in HCM. These findings are not present in physiological, adaptive, hypertrophy of athletes. Therefore, in athletes, any sign of LV hypertrophy coupled with significantly reduced strain values should be evaluated carefully and considered as a potential true HCM and not only physiological adaptation.^34^

### Hypertrophy in infiltrative disease: Amyloidosis

In systemic light chain amyloidosis (AL) - due to a clonal plasma cell disorder - the heart is involved in 50% patients; the symptom progression is most commonly rapid and mortality higher compared to other types of amyloidosis.^35^ In cardiac transthyretin amyloidosis (TTR) - due to amyloid fibrils from liver-derived transthyretin - the heart is involved less frequently and heart symptoms occur at older ages.^1^ Deposits induce oxidant stress and lead to myocardial necrosis and interstitial fibrosis resulting in systolic and diastolic dysfunction.^10^

**Figure 8.**
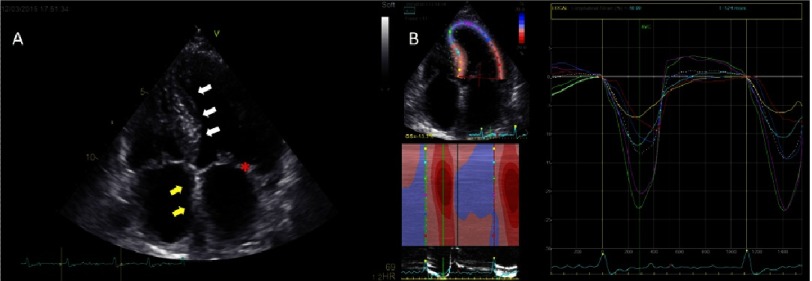
A 64-year-old male with cardiac amyloidosis. (A) The myocardium has a granular appearance. There is hypertrophy of the inferoseptum (*white arrows*) with a normal LV cavity size and notable mitral thickening (red *star*). The atria are enlarged with a thickened interatrial septum (*yellow arrows*). (B) The peak-systolic longitudinal strain values are preserved in the apical segments, while a reduction can be seen in basal and midbasal segments. Global longitudinal strain is reduced.

In cardiac amyloidosis, the myocardium has a granular appearance. There is progressive hypertrophy of both ventricles usually coupled with a normal LV cavity size, whereas the atria are bilaterally enlarged with a thickened interatrial septum due to the restrictive ventricular physiology. Thickening of the mitral and tricuspid valves may also be seen (Figure 8A). A study involving 263 patients with TTR amyloidosis showed that 79% had asymmetrical LVH.^36^

Morphologic traits raise suspicion, but are not sensitive or specific enough to make a diagnosis. A reduction in longitudinal systolic strain can be seen globally, though most prominently in the basal segments (Figure 8B). Commonly, the cardiac apex is the last to be affected, which is often referred to as ‘apical sparing’ - a strain pattern highly suggestive of amyloidosis with reduced longitudinal strain at basal and mid ventricular segments and more preserved at the apical segments (Figure 2C). High values of the relative regional strain ratio (average apical strain/(average basal strain + average mid strain)), seem to be a sensitive and specific marker of cardiac amyloid involvement and are strongly predictive of mortality or need for transplantation.^37^ *Buss et al.*^38^ describe reduced LV longitudinal function assessed by GLS to be an independent predictor of survival in AL amyloidosis (survivors vs. nonsurvivors −13.1 ± 5.4% vs. −9.8 ± 4.4%). Using deformation imaging, LV dysfunction can be detected prior to any signs of morphological or functional impairment as assessed by 2D-echocardiography or assessment of diastolic function.^39^

### Hypertrophy in metabolic disease: Anderson-Fabry disease

Anderson-Fabry disease (AFD) is a lysosomal storage disease caused by an X-linked disorder resulting in deficiency of the enzyme alpha-galactosidase A. This deficiency leads to intracellular globotriaosylceramide (Gb3) accumulation in tissues throughout the body - in the heart, kidneys, central nervous system amongst others - resulting in a variable, often non-specific, combination of signs and symptoms.^40^ Alongside renal disease and stroke, heart disease has become the leading cause of mortality in affected individuals, usually manifesting as mitral and aortic leaflet thickening coupled with regurgitation, LV hypertrophy, diastolic dysfunction, arrhythmias, heart failure, and sudden cardiac death.^41^

Disease severity depends on enzyme activity, which differs in two major phenotypes of the disease. In classic AFD the enzyme activity is absent or below 5% resulting in symptoms beginning in early childhood, whilst cardiac involvement becomes relevant between 20 and 40 years of age.

Lysosomal Gb3 deposits make up only 3% of the mass of the hypertrophic heart, however, the deposition induces oxidative stress and inflammation resulting in hypertrophy and remodelling of the extracellular matrix.^40^ Similarly, as seen in other aetiologies of LV hypertrophy, in a majority of patients with AFD the systolic function is preserved when assessed by EF.^42^ In addition, only 40% of patients have LV hypertrophy at the time of diagnosis.^43^ Therefore assessment of cardiac involvement is challenging with conventional echocardiography. Deformation imaging can detect subclinical impairment of contractile ability before LVH occurs^44^ and help monitor the efficiency of treatment. In the early stage of disease, in patients without high levels of myocardial fibrosis, enzyme replacement therapy proved to be effective in reducing Gb3 accumulation in the myocardium, reducing LV mass and improving cardiac function assessed by deformation imaging.^45^

**Figure 9.**
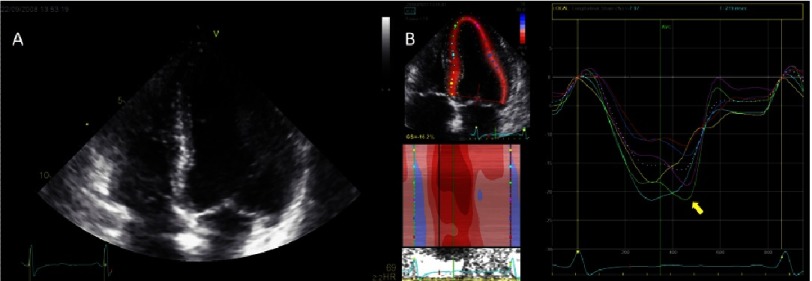
A 53-year-old female with Anderson-Fabry disease. (A) There is a visible hypertrophy in the basal posterior septum, a small left ventricle cavity, and bilateral atrial enlargement. (B) Peak-systolic longitudinal strain is reduced in all myocardial segments which is reflected in the notably low GLS. There is visible post-systolic shortening (*yellow arrow)* and reduced longitudinal deformation particularly involving the inferolateral segments (*blue and red traces*)*.*

On a CMR study with a sample of 13 AFD patients, De Cobelli et al.^46^ observed a consistent pattern of myocardial fibrosis characterized by a mid-myocardial distribution sparing the sub-endocardium, most commonly involving the inferolateral basal or mid-basal segments - findings potentially specific to AFD. Deformation imaging can also depict the typical pattern of distribution of lower longitudinal strain mainly involving the inferolateral segments of the LV. Moreover, several studies^47, 48^ demonstrated that while longitudinal strain was impaired in both AFD and HCM (Figure 9), circumferential strain was significantly lower in AFD than in HCM, in AFD patients with LV hypertrophy and in those without. Both studies demonstrated a loss of the base-to-apex circumferential gradient AFD patients, the gradient being significantly lower as compared to HCM patients. This may be explained in part by the typical midmyocardial distribution of fibrosis in AFD cardiomyopathy since fibre orientation in the midmyocardium determines contribution mainly to circumferential contraction.

## Conclusion: Deformation imaging as an aid to diagnosis

There is a wide spectrum of differential diagnoses for hypertrophic ventricles Different pathologies result in different timing and distribution of cardiac involvement, resulting in different patterns of regional impairment. Deformation imaging contributes to the identification of this underlying aetiology through the visualization of disease specific strain patterns.
